# Neuropsychiatric symptoms and subsyndromes in patients with different stages of dementia in primary care follow-up (NeDEM project): a cross-sectional study

**DOI:** 10.1186/s12877-022-02762-9

**Published:** 2022-01-22

**Authors:** Victoria García-Martín, M. Canto de Hoyos-Alonso, Gloria Ariza-Cardiel, Rosalía Delgado-Puebla, Paula García-Domingo, Erika Hernández-Melo, Javier López de Haro-de Torres, Isabel del Cura-González

**Affiliations:** 1 PhD student in Epidemiology and Public Health at Universidad Rey Juan Carlos (Rey Juan Carlos University), Madrid, Spain; 2 Navalcarnero Health Care Center, Navalcarnero, Primary Care Management, Madrid Health Service, Madrid, Spain; 3 Pedro Laín Entralgo Health Care Center, Alcorcón, Primary Care Management, Madrid Health Service, Madrid, Spain; 4 Red de Investigación Cooperativa Orientadas a Resultados en Salud (RICORS) ISCIII, Madrid, Spain; 5 Family and Community Medicine Teaching Unit Oeste, Primary Care Management, Madrid Health Service, Móstoles, Madrid, Spain; 6 Horta Healthcare Center, Barcelona, Primary Care Management, Catalonia Health Service, Catalonia, Spain; 7 Alcorcón Foundation University Teaching Hospital, Madrid Health Service, Madrid, Spain; 8 Villaviciosa de Odón Health Care Center, Villaviciosa de Odón, Primary Care Management, Madrid Health Service, Madrid, Spain; 9 Research Unit, Primary Care Management, Madrid Health Service, Madrid, Spain; 10 Department of Medical Specialties and Public Health, Universidad Rey Juan Carlos (Rey Juan Carlos University), Alcorcón, Madrid, Spain; 11 Health Services Research on Chronic Patients Network (REDISSEC) ISCIII, Madrid, Spain

**Keywords:** Dementia; Alzheimer’s disease, Behavioural symptoms, Neuropsychiatric symptoms, Prevalence subsyndromes, Primary care, Neuropsychiatric inventory, Global deterioration scale, Psychological tests

## Abstract

**Background:**

The objective was to describe the prevalence and intensity of neuropsychiatric symptoms (NPSs) isolated and grouped into subsyndromes in patients with dementia in primary care (PC) to analyse their distribution based on stages of dementia and the relationship between them and the intensity of symptoms.

**Methods:**

Design: Cross-sectional study. Setting and population: Patients with dementia, not institutionalized, in a PC follow-up. Variables: Sociodemographic and clinical variables. Assessment instruments: The frequency and intensity of NPSs were measured with the Neuropsychiatric Inventory (NPI), and the stages of dementia with the Global Deterioration Scale (GDS). Statistical analysis: The number of NPSs per patient, the mean NPI value, and the prevalence and intensity of NPSs isolated and grouped into subsyndromes were calculated, as were their 95% confidence intervals (CIs). The analyses were performed on an overall basis and by GDS scores. To analyse the association between the NPI and GDS scores, multivariate analysis was performed with a generalized linear model.

**Results:**

Overall, 98.4% (95% CI 94.5;99.8) of the patients presented some type of NPS, with an average of five symptoms per patient. The most frequent symptoms were apathy [69.8% (95% CI 61.1;77.5)], agitation [55.8% (95% CI 46.8;64.5)] and irritability [48.8% (95% CI 39.9;57.8)]. The more intense NPSs were apathy [NPI 3.2 (95% CI 2.5;3.8)] and agitation [NPI 3.2 (95% CI 2.5;4.0)]. For subsyndromes, hyperactivity predominated [86.0% (95% CI 78.8;91.5)], followed by apathy [77.5% (95% CI 69.3;84.4]). By phase of dementia, the most common isolated symptom was apathy (60.7–75.0%). Affective symptoms and irritability predominated in the initial stages, and psychotic symptoms predominated in advanced stages. The mean NPI score was 24.9 (95% CI 21.5;28.4) and increased from 15.6 (95% CI 8.2;23.1) for GDS 3 to 28.9 (95% CI 12.6;45.1) for GDS 7. Patients with in the most advanced stages of dementia presented an NPI score 7.6 (95% CI 6.8;8.3) points higher than the score for mild dementia with adjustment for the other variables.

**Conclusions:**

A high prevalence of NPSs was found among patients with dementia treated in PC. Symptoms change and increase in intensity as the disease progresses. Scales such as the NPI allow these symptoms to be identified, which may facilitate more stage-appropriate management.

**Supplementary Information:**

The online version contains supplementary material available at 10.1186/s12877-022-02762-9.

## Background

Dementia is a process that causes disability and dependency in the elderly, generating significant burdens on caregivers and a high cost for society that varies based on country and disease severity [1]. Neuropsychiatric symptoms (NPSs) or behavioural and psychological symptoms of dementia (BPSDs) [2] are a series of symptoms related to altered perception, content of thought, mood and behaviour that can occur in people with dementia, constituting part of how the disease is expressed. Since 2011, NPSs have been considered, along with cognitive and functional impairment, a basic criterion in the diagnosis of dementia or major neurocognitive disorder [3].

NPSs can occur in 50–98% of patients living in the community [4–15] and include depression, anxiety, apathy, agitation, irritability, continuous complaints, delusions, hallucinations, disinhibition and sleep or appetite disturbances, among others. They appear at any stage of the disease [16], even very early on [4, 10, 14], varying in frequency and intensity based on the degree of cognitive impairment and the type of dementia [11, 17, 18]. Thus, compared with Alzheimer’s disease, depression is more frequent in vascular dementia, and delusions and hallucinations are more frequent in Lewy body dementia, manifesting in earlier stages [18, 19]. NPSs worsen the prognosis and accelerate progression to severe dementia and even death [20, 21].

BPSDs also appear more frequently (43%) in patients with mild cognitive impairment (MCI) than in the general population. Their presence is considered a risk factor for MCI without dementia progressing to dementia [22], with an estimated annual transition rate of 25% [23].

In most patients, several of these symptoms can appear simultaneously, grouped into subgroups of symptoms or subsyndromes whose pathogenesis and management may be similar [2].

There are different scales to evaluate NPSs, in isolation, such as the Geriatric Depression Scale and scales to measure aggression or inappropriate sexual behaviour, or together, such as the Neuropsychiatric Inventory (NPI) [24], the Behavioural Pathology in Alzheimer’s Disease Rating Scale (BEHAVE-AD) [25] and the Alzheimer’s Disease Assessment Scale (ADAS) [26].

Few studies on NPSs have been conducted in the context of primary care (PC) [6, 27]; however, at this level of care, most patients with dementia are treated throughout the disease process.

The main objective of this study was to describe the prevalence and intensity of NPSs isolated and grouped into subsyndromes in patients with dementia treated in PC and to analyse their distribution based on stages of dementia. As a secondary objective, the relationship between the stages of dementia and the intensity of symptoms, as measured by the NPI score, was analysed.

## Methods

### Study design, setting and participants

This was a cross-sectional descriptive observational study in two urban health centres in the municipalities of Alcorcón and Villaviciosa de Odón located in the western portion of the Community of Madrid (Spain); these municipalities have a combined registered population of 43,594 people, of whom 9247 were ≥ 65 years old. For the preparation of the article, the STROBE recommendations were followed [28].

Between November 1, 2015, and January 31, 2016, patients of all ages with a previous diagnosis of dementia identified with the International Classification of Primary Care (ICPC) code P70 and/or with specific treatment for dementia (anticholinesterase drugs (ATC code: N06D) and/or memantine (ATC code: N06DX01)) were selected from the electronic health records (EHRs) of the Community of Madrid (PC-Madrid). Eligible patients had a least one consultation or received PC in 2015 and had a known caregiver who agreed to participate in the study and signed the informed consent form. For patients with professional caregivers, informed consent was also requested from the legal representative of the patient. Informed consent was also requested from the patient himself or herself if considered able to do so at the discretion of the physician responsible. Institutionalized patients and/or patients with previous major mental disorders such as schizophrenia or other psychotic disorders were excluded, as well as those patients whose caregivers presented difficulties with language while conducting the interview and those who refused to participate in the study.

Based on these criteria, 129 patients were included in the study. With this sample size and considering an estimated prevalence of NPSs from 75 to 98%, based on different published studies [4, 6, 9, 10, 14], the estimation precision for our study was between 2.4 and 7.5%.

Data collection was performed by reviewing the EHRs of the patients and interviewing primary caregivers.

### Variables and assessment instruments

The following patient sociodemographic variables were collected: age, sex, highest level of education, type of coexistence and relationship between patient and caregiver. The following clinical variables were collected: duration of dementia, cognitive function and progression stage, functional assessment, presence of NPSs and treatment for dementia (specific and for NPSs). Reisberg’s [29] Global Deterioration Scale (GDS) was used to classify the progression stage, where GDS 3 is mild cognitive decline and GDS 7 is very severe cognitive decline. For the analysis, the stages were grouped into mild (GDS 3 and 4), moderate (GDS 5) and severe (GDS 6 and 7) dementia. Functional assessment was performed using the Barthel index [30] with the levels of dependency established by Shah et al. [31]. Dementia-specific treatment was considered if they had been prescribed anticholinesterase drugs (ATC code: N06D) and/or memantine (ATC code: N06DX01). The use of neuroleptics, antidepressants and/or benzodiazepines was assumed to be a possible symptomatic treatment for NPSs.

The frequency and severity of NPSs were measured with the NPI [32], a structured interview whose objective is to obtain information on the presence of psychological and behavioural symptoms in patients with Alzheimer’s disease and other dementias. It explores the presence, in a preset period of time, usually the last month, of 10 different symptoms or subscales: delusions, hallucinations, agitation/aggressiveness, depression, anxiety, elation/euphoria, apathy/indifference, disinhibition, irritability/lability, and aberrant motor behaviour (NPI-10), to which sleep and nighttime behaviour disorders and appetite and eating disorders were added later (NPI-12) (https://eprovide.mapi-trust.org/instruments/neuropsychiatric-inventory-12-item-version). The NPI measures the frequency of each of these symptoms from 1 (less than once per week) to 4 (very frequently) and severity from 1 (produces little distress in the patient) to 3 (very disturbing to the patient and difficult to redirect). The score for each subscale is obtained from the product of the frequency and severity of each specific symptom. The total score is obtained by adding the value for all the subscales and ranges from 0 points (absence of neuropsychiatric disorder) to a maximum of 144 points. It has been validated in the Spanish population [33]. Before data collection, the six researchers who conducted the interviews received prior information and training on the proper use of this tool.

The symptoms detected with the NPI were divided into two groups based on whether they were significant (those whose intensity, that is, the product of frequency and severity, was ≥4) or not significant (intensity score < 4) [18].

In addition, the symptoms detected with the NPI were grouped into four subsyndromes or subgroups of symptoms based on the classification of Aalten et al. 2007 [2]: “hyperactivity” (aggressiveness, disinhibition, irritability, aberrant motor behaviour and euphoria); “psychosis” (hallucinations, delusions and sleep disturbance); “Affective” (depression and anxiety) and “apathy” (apathy and appetite disturbance). The criterion used to define the presence of a subsyndrome was that the patient presented one or more of the symptoms that constituted the group; the presence of all symptoms of a subsyndrome simultaneously was not necessary.

### Statistical analysis

A descriptive analysis of the sociodemographic and clinical characteristics of the included patients was performed. The qualitative variables are expressed as frequencies and percentages, and the quantitative variables are expressed as means and standard deviations or medians and interquartile ranges for data with a nonnormal distribution.

The prevalence of NPSs, the number of symptoms per patient and the mean value of the NPI based on sociodemographic and clinical characteristics were calculated, as were the 95% confidence intervals. The frequency, severity and intensity of NPSs were described, each separately and grouped into subsyndromes [2]. Calculations were performed for all symptoms and for significant symptoms (NPI ≥ 4) [4]. In each GDS progression stage, the mean number of symptoms per patient, the mean value of the NPI and the frequency and intensity of each symptom and of each subsyndrome were analysed.

The association of sociodemographic and clinical variables with significant (NPI ≥ 4) or nonsignificant (NPI < 4) symptoms was assessed using the chi squared test, and associations with the NPI were measured as a total score using Student’s t-test and ANOVA. To analyse the relationship between the NPI score (dependent variable) and the stages of dementia (mild, moderate and severe dementia), a generalized linear model (GLM) was constructed. As fit variables, sociodemographic variables (age, sex, and education) and clinical variables (duration, level of dependency based on the Barthel index, specific treatment for dementia, and treatment with neuroleptics, antidepressants and benzodiazepines) were included. This analysis tool was chosen because of its greater tolerance to not meeting the assumptions necessary to build classical models; GLMs can obtain unbiased estimators of associations in the presence of heteroscedasticity [34]. Maximum likelihood methods were used for parameter estimations in the GLM, allowing us to obtain results without having to smooth the dependent variable and without the possible heteroscedasticity being problematic [35, 36]. To prevent errors in the specification of the model, estimate errors were calculated by robust methods [37, 38] considering the inclusion of patients from different groups (health centres). To select the best model, the Akaike information criterion (AIC), Bayes information criteria (BIC) and the adjusted McFadden pseudo-R^2^ were studied [39]. Improvement in BIC values was assessed based on the interpretations proposed by Kass and Raftery [40].

Statistical analyses were performed with SPSS version 26, STATA version 14 and R studio version 1.4.17.17.

### Ethics approval

This study was conducted following the principles of the Declaration of Helsinki and its subsequent revisions and was approved by the Clinical Research Ethics Committee of Alcorcón Foundation University Hospital on September 23, 2015.

## Results

**Fig. 1:**
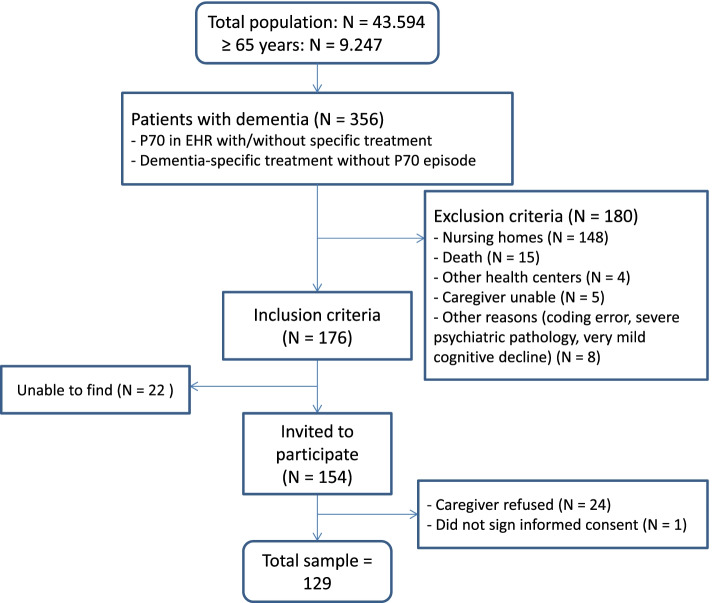
Study flowchart. Legend: P70: ICPC code (International Classification of Primary Care). EHRs: electronic health records. IC: informed consent

Of the 356 patients with dementia identified (ICPC code P70 and/or use of specific treatment for dementia), 176 met the inclusion criteria and 129 agreed to participate in the study. Figure 1 shows the flowchart for the study.

No significant differences were found in terms of sex and age between the participating patients and those who refused to participate or were not located. The mean age of the patients was 82.7 years [8], with a predominance of women (70.5%). A total of 70.5% were older than 80 years, and only two patients were younger than 65 years. Regarding level of education, the majority (72.9%) had primary education (34.1% incomplete), 7.0% were illiterate, and a minority had higher education or university studies (2.4%). The majority lived with a partner (29.4%) or with other relatives, especially their children (51.2%), and 13.2% of the patients lived with a professional caregiver. A total of 62.8% had more than 3 years of cognitive decline, and more than half (52.7%) had severe or total functional dependency with respect to basic activities of daily living. Considering cognitive function and stage of progression of dementia, most patients (83.7%) had a GDS score of 4, 5 or 6 (mild, moderate and severe dementia, respectively), with the initial (GDS 3) and final (GDS 7) stages being less represented. A total of 72.9% were under specific treatment for dementia with anticholinesterase drugs and/or memantine. Of the drugs used for NPSs, 48.1% were antidepressants, 42.6% were neuroleptics, and 35.7% were benzodiazepines. Table 1 shows the main characteristics of the patients who participated in the study.

**Table 1:** Sociodemographic and clinical characteristics of and NPI scores for patients with dementia included in the study

	N (%)	Total NPI score		
		Mean (SD)	CI (95%)	***p***
Age				
< 65 years	2 (1.6%)	10.5 (6.4)	(−46.7;67.7)	0.709
65–74 years	21 (16.3%)	26.2 (23.0)	(15.8;36.7)	
75–79 years	15 (11.6%)	27.4 (27.3)	(12.3;42.5)	
≥ 80 years	91 (70.5%)	24.6 (18.0)	(20.8;28.3)	
Sex				
Men	38 (29.5%)	24.2 (21.8)	(17.0;31.4)	0.782
Women	91 (70.5%)	25.3 (19.2)	(21.3;29.2)	
Education				
Illiterate	9 (7.0%)	20.7 (22.1)	(3.7;37.6)	0.619
No education (less than 5 years)	44 (34.1%)	26.3 (19.5)	(20.4;32.3)	
Primary education (more than 5 years, without completing mandatory education to 16 years old)	50 (38.8%)	27.2 (20.7)	(21.3;33.1)	
Secondary education, 7th-10 grade (EGB, ESO, elementary baccalaureate)	17 (13.1%)	21.6 (20.4)	(11.1;32.1)	
Baccalaureate, 11th–12th grade high school (post-16 education)	6 (4.6%)	17.2 (14.0)	(2.4;31.9)	
Higher education (vocational) and university	3 (2.4%)	14.0 (13.5)	(−19.6;47.6	
Habitation				
Alone	8 (6.2%)	19.3 (20.6)	(2.0;36.5)	0.609
With partner	38 (29.4%)	25.3 (21.0)	(18.4;32.2)	
With family (with or without partner)	66 (51.2%)	24.1 (18.5)	(19.6;28.7)	
With professional caregiver	17 (13.2%)	29.9 (23.1)	(18.1;41.8)	
GDS stage				
GDS 3 (mild CD, borderline deterioration)	8 (6.2%)	15.6 (8.9)	(8.2;23.1)	0.301
GDS 4 (moderate CD, mild dementia)	38 (29.4%)	21.0 (16.6)	(15.5;26.4)	
GDS 5 (moderately severe CD, moderate dementia)	42 (32.6%)	27.1 (22.3)	(20.1;34.0)	
GDS 6 (severe CD, moderately severe dementia)	28 (21.7%)	28.0 (18.1)	(20.9;35.0)	
GDS 7 (very severe CD, severe dementia)	13 (10.1%)	28.9 (26.9)	(12.6;45.1)	
Barthel index				
Independent (100 points)	18 (14.0%)	14.7 (14.0)	(7.8;21.7)	0.214
Slight dependency (91–99 points)	6 (4.6%)	22.7 (27.5)	(−6.2;51.5)	
Moderate dependency (61–90 points)	37 (28.7%)	26.2 (20.1)	(19.5;33.0)	
Severe dependency (21–60 points)	35 (27.1%)	27.0 (17.6)	(21.0;33.0)	
Total dependency (< 21 points)	33 (25.6%)	27.3 (22.5)	(19.3;35.2)	
Duration of dementia				
≤ 1 year	9 (7.0%)	30.8 (25.3)	(11.3;50.2)	0.736
1–3 years	39 (30.2%)	25.6 (21.8)	(18.5;32.7)	
3–6 years	47 (36.5%)	23.0 (15.4)	(18.4;27.5)	
6–9 years	15 (11.6%)	28.7 (25.3)	(14.7;42.7)	
More than 9 years	19 (14.7%)	22.8 (19.4)	(13.4;32.2)	
Treatment				
No specific treatment	35 (27.1%)	26.4 (22.2)	(18,8;34.0)	0.613
Specific treatment: CEI and/or memantine	94 (72.9%)	24.4 (19.1)	(20.5;28.3)	
Treatment with neuroleptics				
No	74 (57.4%)	19.7 (16.5)	(15.9;23.6)	0.001
Yes	55 (42.6%)	31.9 (22.0)	(26.0;37.9)	
Treatment with benzodiazepines				
No	83 (64.3%)	24.0 (20.7)	(19.5;28.5)	0.469
Yes	46 (35.7%)	26.7 (18.5)	(21.2;32.2)	
Treatment with antidepressants				
No	67 (51.9%)	21.3 (17.7)	(17.0;25.7)	0.034
Yes	62 (48.1%)	28.8 (21.5)	(23.4;34.3)	

*NPI* Neuropsychiatric Inventory, *GDS* Global Deterioration Scale, *CD* cognitive decline, *CEI* cholinesterase inhibitors

A total of 98.4% (95% CI 94.5; 99.8) of the patients had some neuropsychiatric symptoms, and 84.5% (95% CI 77.1; 90.3) had at least one symptom of clinically significant intensity (NPI ≥ 4).

The mean number of symptoms per patient was 5 (95% CI 4.6; 5.5), decreasing to 3 (95% CI 2.5; 3.3) when considering only symptoms of significant intensity. The mean total NPI score (per patient) was 24.9 (95% CI 21.5; 28.4), with a median of 21 (IQR: 10.8–34.0), with the highest scores for patients who were treated with neuroleptics or antidepressants (*p* < 0.05), drugs used for the treatment of these symptoms. (See Table 1 and Additional file 1).

A relationship was observed between the stage of dementia (GDS) and the NPI score, increasing by 7.6 points on average (95% CI 6.8; 8.3) in the presence of severe dementia versus mild dementia, after adjusting for sex, age, duration of dementia, and treatment with neuroleptics and antidepressants (See Table 2).

**Table 2:** Relationship between the Neuropsychiatric Inventory (NPI) score and the stages of dementia based on the Global Deterioration Scale (GDS)

Total NPI score	Coef	Robust Std. Err.	p	95% CI
Age				
< 65 years	ref			
65–74 years	9.64	1.09	0.000	7.50; 11.77
75–79 years	10.02	5.98	0.094	- 1.70; 21.74
≥ 80 years	7.32	0.48	0.000	6.38; 8.26
Sex (men/women)				
Women	ref			
Men	- 0.42	2.96	0.887	- 6.23; 5.39
GDS stage				
Mild dementia (GDS 3 and 4)	ref			
Moderate dementia (GDS 5)	8.30	5.78	0.151	- 3.03; 19.64
Severe dementia (GDS 6 and 7)	7.58	0.38	0.000	6.83; 8.32
Duration of dementia				
≤ 3 years	ref			
> 3 years	−5.01	1.43	0.000	- 7.81; −2,22
Treatment with neuroleptics				
No	ref			
Yes	11.22	5.07	0.027	1.29; 21.16
Treatment with antidepressants				
No	ref			
Yes	6.44	0.40	0.000	5.65; 7.22

General linear model. Family: Gaussian. Linking function: identity

AIC: 1115.44

BIC: 1118.30

McFadden pseudo-R^2^ adjusted: 15.5%

*NPI* Neuropsychiatric Inventory, *GDS* Global Deterioration Scale

### Prevalence and intensity of neuropsychiatric symptoms and subsyndromes

**Fig. 2:**
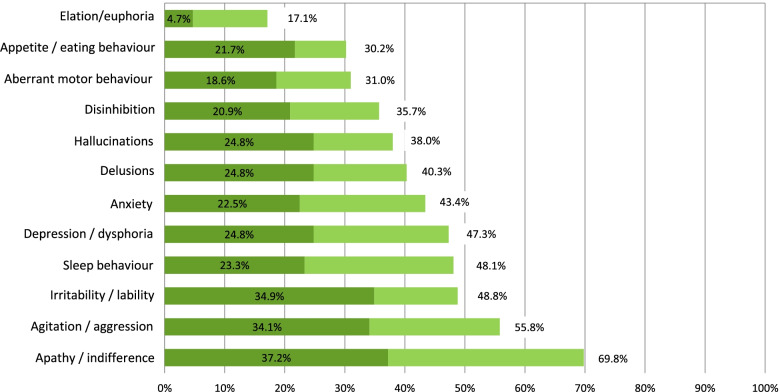
Prevalence rates of total neuropsychiatric symptoms and clinically significant symptoms (NPI score ≥ 4) in patients with dementia included in the study. Legend: Light bars represent the prevalence of total symptoms, and dark bars the prevalence of clinically significant symptoms (those with an NPI frequency by severity score ≥ 4)

The most frequent symptom was apathy [69.8% (95% CI 61.1; 77.5)], followed by agitation [55.8% (95% CI 46.8; 64.5]). Symptom intensity calculated by frequency and severity was greater for both apathy, with a mean of 3.2 (95% CI 2.5; 3.8), and agitation, with a mean of 3.2 (95% CI 2.5; 4.0), but the symptom that the caregivers considered the most severe (symptoms scoring a 3 for severity according to the NPI scale, highly bothersome to the patient and difficult for the caregiver to manage) was agitation [18.6% (95% CI 12.3; 26.4)]. Euphoria was the least frequent [17.1% (95% CI 11.0; 26.7)], lowest severity [1.6% (95% CI 0.2; 5.5)] and lowest intensity symptom [0.5 (95% CI 0.3; 0.8)]. When considering only significant symptoms (NPI ≥ 4), the most frequent were apathy [37.2% (95% CI 28.9; 46.2)], irritability [34.9% (95% CI 26.7; 43.8)] and agitation [34.1% (95% CI 26.0; 43.0)], and the highest intensity symptoms were agitation [8.4 (95% CI 7.5; 9.4)], sleep disorders [8.1 (95% CI 7.0; 9.3)] and hallucinations [7.8 (95% CI 6.7; 8.9)] (See Fig. 2, Additional file 2 and Additional file 3).

**Fig. 3:**
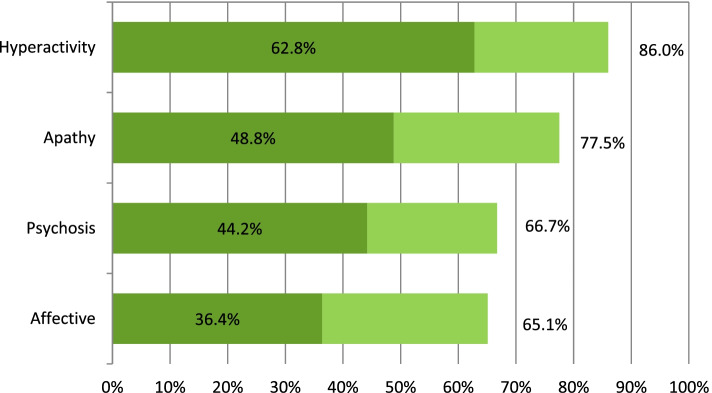
Frequency of total neuropsychiatric subsyndromes and clinically significant symptoms (NPI ≥ 4), in patients with dementia included in the study. Legend: Hyperactivity subsyndrome: aggressiveness, disinhibition, irritability, aberrant motor behaviour and euphoria. Apathy subsyndrome: apathy and appetite disorders. Psychosis subsyndrome: hallucinations, delusions and sleep disorders. Affective subsyndrome: depression and anxiety. Light bars represent the prevalence of subsyndromes with total symptoms, and dark bars represent those with clinically significant symptoms (those with an NPI frequency by severity score ≥ 4)

When grouping symptoms by subsyndromes [2], the most common was hyperactivity both in overall frequency [86.0% (95% CI 78.8; 91.5)] and in significant symptomatology [62.8% (95% CI 53.8; 71.1)]. Psychotic and affective symptoms were presented in similar proportions ([66.7% (95% CI 57.8; 74.7)] and [65.1% (95% CI 56.2; 73.3)], respectively), although psychotic symptoms contributed to a greater proportion of significant symptoms [44.2% (95% CI 35.4; 53.2)] (see Fig. 3 and Additional file 2). When analysing the intensity of the subsyndromes, those with the highest mean value were hyperactivity [9.4 (95% CI 7.8; 11.0)] and psychosis [6.5 (95% CI 5.5; 8.1)] (see Additional file 3).

### Neuropsychiatric symptoms and subsyndromes based on the developmental stage of dementia

**Fig. 4:**
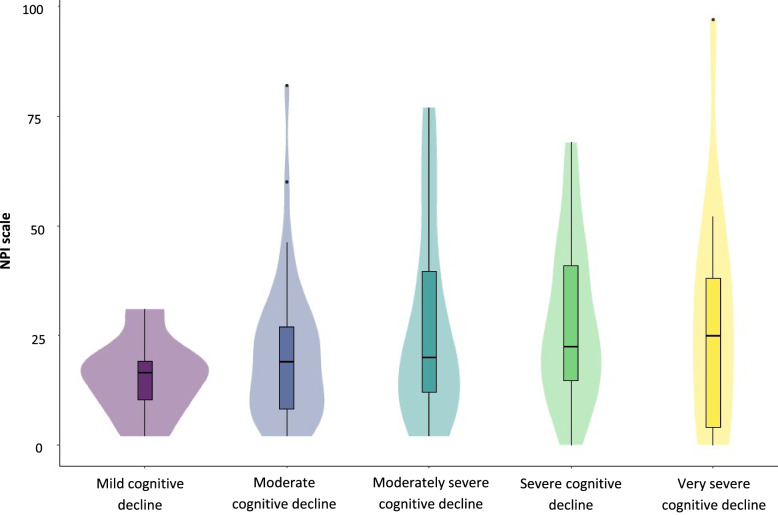
The mean NPI score based on dementia progression (GDS stage)

Figure 4 shows the distribution of (the median) NPI in the different stages or phases of dementia based on the GDS classification. The mean NPI score for GDS 3 was 15.6 (95% CI 8.2; 23.1), and that for GDS 7 was 28.9 (95% CI 12.6; 45.1) (see Table 1 and Fig. 4).

**Fig. 5:**
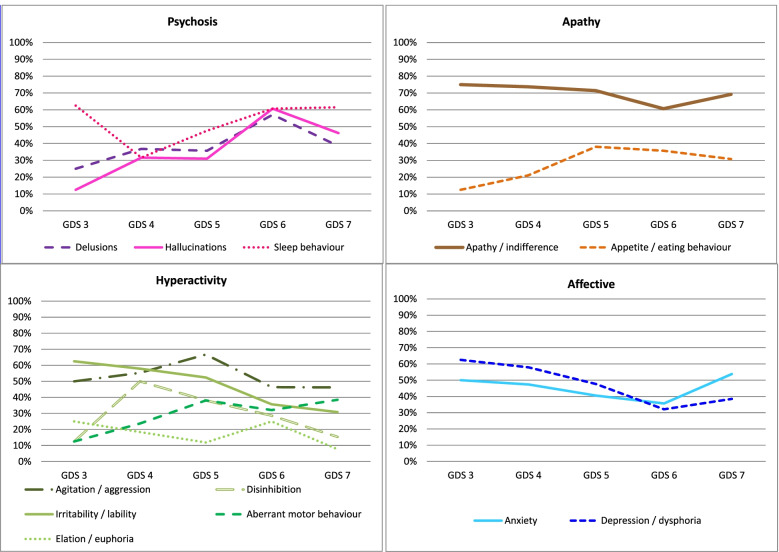
Frequency of neuropsychiatric symptoms based on dementia progression (GDS stage) grouped by subsyndromes [2]. Legend: GDS: Global Deterioration Scale

**Fig. 6:**
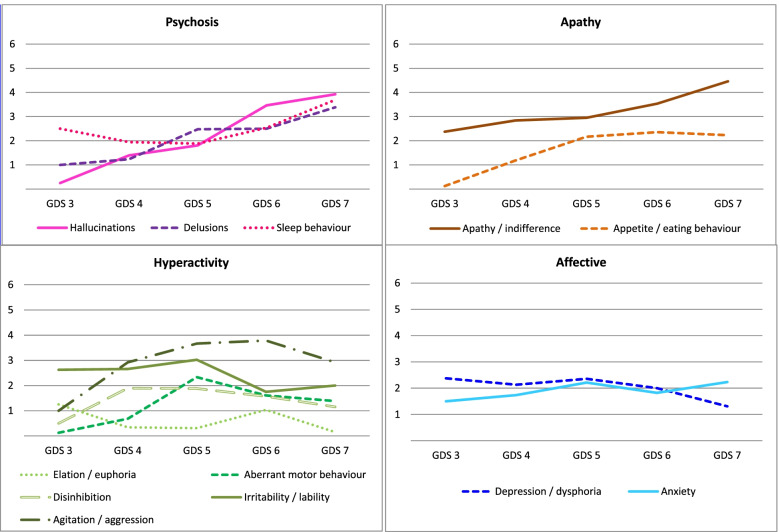
Intensity of neuropsychiatric symptoms in each developmental stage of dementia (GDS) grouped by subsyndromes [2]. Legend: Intensity: the average frequency and severity scores for each symptom; range of 0 to 12 for all symptoms. GDS: Global Deterioration Scale

When analysing the distribution of symptoms based on the stages of dementia, variation was observed as cognitive deterioration advanced. Apathy remained the most frequent symptom in all phases of the disease, occurring in 60.7 to 75.0% of patients. In early stages, depression, anxiety and irritability predominated, present in more than 50% of patients. Psychotic symptoms (delusions, hallucinations) were more common in more advanced stages, GDS 6 and 7. The intensity of the symptoms as deterioration progressed was also not homogeneous. Anxiety and depression remained more stable, while the intensity of apathy, hallucinations and delusions increased progressively throughout the disease. Statistical significance was only found for hallucinations (see Fig. 5, Fig. 6 and Additional file 4).

The distribution of clinically significant symptoms (NPI ≥ 4) in the different phases of dementia in terms of their frequency and intensity was similar to that described for the overall symptoms, and the trend was also significant only for hallucinations (See Additional file 5).

**Fig. 7:**
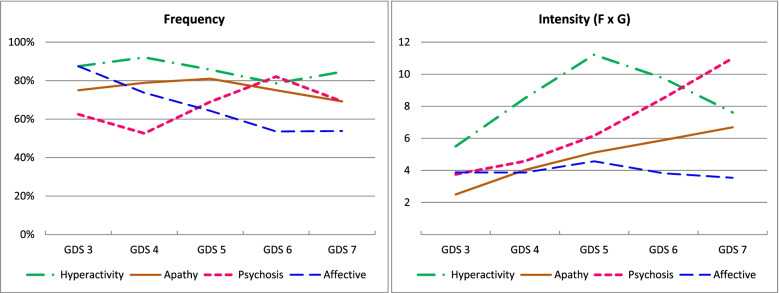
Frequency and intensity of neuropsychiatric subsyndromes based on the GDS stage of dementia. Legend: GDS: Global Deterioration Scale

When grouping by subsyndrome, the frequency of hyperactivity and apathy remained relatively stable throughout progression, with hyperactivity being the most frequent subsyndrome in all stages except in GDS 6, during which psychotic symptoms predominated [82.1% (95% CI 63.1; 93.9)]. The affective subsyndrome was more common in mild [87.5% (95% CI 47.3; 99.7)] or moderate cognitive decline [73.7% (95% CI 56.9; 86.6)], and its frequency decreased as the disease progressed. For intensity, as the disease progressed, the mean value of the subsyndromes apathy and psychosis increased, with maximum values of 6.7 (95% CI 2.5; 10.9) and 11 (95% CI 3.4; 18.6), respectively, for GDS 7, the affective subsyndrome remained stable, and hyperactivity was more intense in intermediate phases, with values up to 11.2 (95% CI 8.0; 14.5) for GDS 5 (see Fig. 7 and Additional file 6).

## Discussion

The prevalence of NPSs found in our study was very high and tended to be highest in the more advanced stages of dementia that present worse functionality, with different NPSs presenting different disease severity.

According to their caregivers, almost 100% of the patients presented some neuropsychiatric symptoms, and approximately 85% had at least one clinically significant symptom, that is, an NPI score ≥ 4. The prevalence results obtained in our study in noninstitutionalized patients are similar to those reported by other studies carried out in specialized clinics, with prevalence rates exceeding 90% [8–11], but higher than those reported for community-dwelling patients, with an overall NPS prevalence of 50–85% [5, 6, 12–14] and significant symptom prevalence of 40–67% [4, 12, 18, 41]. The higher prevalence of NPSs found can be explained because patients previously diagnosed with dementia were included, unlike other studies that recruited patients by screening for dementia in the general population, possibly leading to a higher percentage of cases of mild dementia [5, 12].

Our patients presented an average of five neuropsychiatric symptoms. Studies using the same 12-item version of the NPI have reported similar findings [6, 9]. Apathy was the most frequent NPS, appearing in 70% of patients and being clinically relevant in almost 40%. These data are consistent with those of other studies, with prevalence rates between 74 and 76% [9, 42, 43]. Apathy stands out as the most common symptom in most publications [8–10, 14, 15], but depression [7, 44–46], sleep [12] and appetite disturbances [13] have also been described as the most prevalent. These differences may be related to the baseline characteristics of the study population. Thus, depression predominated when the percentage of mild [7, 45, 46] or moderate [44] dementia in patients was high. Although apathy was the most frequent symptom, agitation, sleep disorders and hallucinations were the most intense and had the greatest weight in the overall NPI score due to the impact that they had on the patients and their caregivers.

The average total NPI score was 25, without differences based on sex or age group, as noted in other publications [12, 46]. We also did not find differences based on level of education or level of dependency. Regarding coexistence, the NPI score was higher for patients who lived with a professional caregiver than for those who lived alone or with family (partner or other relatives). Although this association was not statistically significant, it reflects the reality of having to hire outside caregivers for individuals with a greater intensity of symptoms. The average NPI score was very similar to the scores reported in studies by Aalten et al. 2007 [2] (mean NPI 23) and García-Alberca et al. 2008 [9] (mean NPI 27.9); in contrast, the mean score obtained herein was higher than the 12–15 points obtained by other authors, a difference that could be explained by different characteristics of the study populations, with a predominance of mild dementia, or the use of a 10-symptom version of the NPI instead of the NPI-12 [27, 44, 46, 47].

The NPI score (NPS intensity) tended to increase as the disease progressed, a relationship previously described in other studies [15, 18, 48, 49]. In our study, an increase of 7.6 points in the NPI was demonstrated between mild and advanced dementia after adjusting for sex, age and clinical factors that could influence this score, such as the duration of dementia and symptomatic treatment of NPSs with neuroleptics or antidepressants. In terms of the distribution of symptoms based on the different stages, apathy was the most frequent in all stages. In the early stages (GDS 3), depression, anxiety, irritability and sleep disorders predominated, as observed in other studies in which symptoms were analysed in patients with mild cognitive decline or mild dementia [10, 14, 15, 45]. Agitation became more frequent in GDS stages 4 and 5, which correspond to mild-moderate dementia, and psychotic symptoms (delusions and hallucinations) were more common in advanced stages (GDS 6 and 7), as observed in other studies [15, 42].

Changes in the frequency and intensity of NPSs as the disease progresses can be better observed by grouping symptoms into subsyndromes [2]. The frequency of NPSs remained stable for the hyperactivity and apathy subsyndromes, decreased for the affective subsyndrome and increased for the psychotic subsyndrome. In contrast, intensity behaved differently, increasing both for the psychotic subsyndrome and for apathy throughout disease progression, and was more homogeneous for the affective and hyperactivity subsyndromes.

Numerous studies have grouped NPSs into subsyndromes [13, 15, 21, 27, 45, 47, 50, 51]; however, few studies have examined their distribution based on GDS stage [52], especially in community-dwelling patients. This grouping by subsyndrome is of great interest from the clinical point of view, especially in a scenario of high frequency consultations, as happens in PC, because it allows simplification of the detection of NPSs and provides a more suitable approach with pharmacological or nonpharmacological measures based on the predominating symptom at any given time [53–56].

Among the limitations, notably, although the size of our sample was small compared with those in population-based studies [7, 11, 15, 18, 57], the sample size is consistent with those in other studies performed in clinical practice [8–10, 14, 27, 41]. One strength is that our entire eligible population was included as study subjects. We believe that our work with noninstitutionalized patients in PC follow-up allows a better approach for community-based patients than approaches performed in neurology and geriatric consultations [2, 7–11, 15, 16, 18, 41] with more restrictive selection criteria or in institutionalized patients [49, 58] who have more advanced stages of dementia.

In our study, the cognitive level and stage of progression were determined using the GDS by questioning the caregiver. Interviewing or administering cognitive tests to the patient directly was not considered following the recommendations of the Clinical Research Ethics Committee (CEIm), which suggests limiting patient distress as much as possible if interviewing the caregiver can answer the research question. When available, the Mini-mental State Examination (MMSE) was also used, but it was not included in the final analysis because these data were not available for all patients, and it was not essential for staging.

Unlike other studies that only included individuals with mild-moderate stages of dementia [9, 10, 14] or that limited the age of inclusion [6, 7, 12, 13], in our study, we included patients at all stages of dementia of any age and with a diagnosis of dementia of any aetiology. We believe that this approach provides greater external validity to our results, although it may limit comparisons with other studies that only study Alzheimer’s disease [10, 11, 15, 41] and/or the most frequent dementias [7, 18]. For example, the wide dispersion of symptoms that we observed in advanced stages may be related to some patients who presented rapid-progression non-Alzheimer dementia.

Although patients in any stage of dementia were included, the representativeness of the initial and final stages was low. The latter could have occurred because a high percentage of these individuals are treated in nursing homes, which usually occurs for more advanced dementia and/or dementia with more intense symptoms. The lower representativeness of the initial stages could be explained by the usual delay in confirming the diagnosis from the onset of the first symptoms of dementia [59]. Patients with memory deterioration or very mild cognitive decline could have early dementia but were not included, as only patients with confirmed dementia were included in the study. Accordingly, the results obtained in our study can be generalized to noninstitutionalized patients with a registered diagnosis of dementia treated in PC, which limits their external validity to this setting. The institutionalized population with dementia was not the object of study. In relation to the generalization of the results in the PC field, the fact that mild dementia may be underrepresented in our sample due to registration issues in the initial stages, especially until a definitive diagnosis is assigned, warrants special consideration. This lack of registration or underdiagnosis, which mainly affects patients with mild dementia, has been reflected in other studies carried out in PC in Spain [60, 61] and in other countries [59] and indicate the need to implement institutional measures that facilitate an earlier diagnosis of patients with dementia, from adequate training of health professionals to agile systems allowing the execution of diagnostic tests in PC and/or referral of patients without excessive delays or other levels of care, if necessary, to complete the diagnosis.

Despite the need for population studies to determine the true prevalence of dementia, the cost and duration of these studies does not allow them to be carried out in all types of populations or to update them continuously. Therefore, having other more agile detection systems that allow periodic estimation of the prevalence of dementia without excessive error is advisable. In this sense, for estimation of patients with dementia through real-world data, our method of obtaining data, which has been demonstrated in other studies conducted in Spain [62, 63], adequately reflects reality by yielding results that fall within the margins found in population prevalence studies conducted both in our environment [64–66] and in Europe and the United States [67, 68].

This study is of interest because of the importance that these symptoms have in the management of the disease and the few studies on NPSs performed at this level of care [6, 27]. NPSs condition the quality of life of the patient and caregiver and are one of the main reasons for PC consults by caregivers of patients with dementia. Knowing the frequency and intensity of the most significant symptoms and their course in the disease is crucial for patient management. However, in clinical practice, objective measurement instruments are not usually used to identify symptoms or to monitor treatment regimens. They are also not usually the object of research in PC, unlike the scales that measure cognitive symptoms (such as the MMSE) or function (such as the Barthel or Lawton activities of daily living scales), commonly used both in clinical practice and research. We believe that to improve the approach to these symptoms and the adequate care of patients and caregivers, it is essential to have a perspective from the reality of PC, reinforcing research in this field. Another point of interest is to verify whether having more financial means and/or institutional support for care (possibility of hiring a professional caregiver and/or help in day centres) reduces the burden on caregivers or delays the institutionalization of patients with dementia. Making this issue evident may facilitate more public assistance to patients with dementia and their families.

## Conclusions

The prevalence of NPSs estimated with the NPI scale in noninstitutionalized patients with dementia in PC follow-up is high and changes based on the different progression stages of dementia, with an upward trend in the NPI score as dementia progresses. The most frequent and intense symptoms are apathy and agitation. As dementia progresses, the frequency of apathy is maintained, but its intensity increases. Psychotic symptoms (delusions, hallucinations) increase in frequency and intensity, and affective symptoms (depression, anxiety) decrease in frequency and maintain a similar intensity at all stages. The grouping of symptoms in subsyndromes (apathy, hyperactivity, psychotic and affective symptoms) helps to better illustrate the differences in the patterns of symptoms throughout the disease and can improve the guidance provided to caregivers regarding how to management their patient based on which subsyndrome predominates at each progression stage.

## Supplementary Information

**Additional file 1.** Distribution of neuropsychiatric symptoms by sex, age group and GDS stage based on the intensity of symptoms.

**Additional file 2.** Prevalence of neuropsychiatric symptoms and subsyndromes in patients with dementia included in the study.

**Additional file 3.** Intensity of symptoms and neuropsychiatric subsyndromes in patients with dementia included in the study.

**Additional file 4.** Frequency and intensity of neuropsychiatric symptoms based on the progression of dementia (GDS stage).

**Additional file 5.** Frequency and intensity of significant neuropsychiatric symptoms (NPI ≥ 4) based on the progression of dementia (GDS stage).

**Additional file 6.** Frequency and intensity of neuropsychiatric subsyndromes based on the progression of dementia (GDS stage).

## Data Availability

The datasets used and/or analysed during the current study are available from the corresponding author on reasonable request. All data generated or analysed during this study are included in this published article [and its supplementary information files].

## Abbreviations

ATC: Anatomical therapeutic chemical classification system
PC: Primary care
IC: Informed consent
ICPC: International classification of primary care
CD: Cognitive decline
EHR: Electronic health record
CEI: Cholinesterase inhibitor
GDS: Global Deterioration Scale
MMSE: Mini-Mental State Examination
NPI: Neuropsychiatric Inventory
P70: ICPC dementia code
BPSDs: Behavioural and psychological symptoms of dementia
NPSs: Neuropsychiatric symptoms
STROBE: Strengthening the Reporting of OBservational Studies in Epidemiology
