# Supplementary material for: Neuropsychiatric symptoms and subsyndromes in patients with different stages of dementia in primary care follow-up (NeDEM project): a cross-sectional study
Source: BMC Geriatr. 2022 Jan 22;22:71. doi: 10.1186/s12877-022-02762-9 (PMC8783993; doi:10.1186/s12877-022-02762-9)
Supplement: Supplementary file 1 — Additional file 1. Distribution of neuropsychiatric symptoms by sex, age group and GDS stage based on the intensity of symptoms. [file 12877_2022_2762_MOESM1_ESM.docx]

Appendix 1 Distribution of neuropsychiatric symptoms by sex, age group and GDS stage based on the intensity of the symptoms

|  | **Total patients**  **(N=129)** | **Patients with non-significant symptoms (NPI < 4) (N = 20)** | **Patients with significant symptoms (NPI ≥ 4) (N = 109)** |
| --- | --- | --- | --- |
| **Percentage of patients with some type of symptom** ^1^ | 98.4% (94.5;99.8) | 15.5% (9.7;22.9) | 84.5% (77.1;90.3) |
| - **Sex** |  |  |  |
| Men (N=38) | 97.4% (86.2;99.9) | 13.2% (4.4;28.1) | 86.8% (71.9;95.5) |
| Women (N=91) | 98.9% (94.0;99.9) | 16.5% (9.5;25.7) | 83.5% (74.2;90.4) |
| **- Age** |  |  |  |
| < 65 years (N=2) | 100% | 0% | 100% |
| 65-74 years (N=21) | 95.2% (76.2;99.9) | 23.8% (8.2;47.2) | 76.2% (52.8;91.8) |
| 75-79 years (N=15) | 93.3% (68.0;99.8) | 20.0% (4.3;48.1) | 80.0% (51.9;95.7) |
| ≥ 80 years (N=91) | 100% | 13.2% (7.0;21.9) | 86.8% (78.1;93.0) |
| **- GDS** |  |  |  |
| GDS 3 (N=8) | 6.2% (2.7;11.8) | 12.5% (0.3;52.7) | 87.5% (47.3;99.7) |
| GDS 4 (N=38) | 29.4% (21.8;38.1) | 15.8% (6.0;31.3) | 84.2% (68.7;93.9) |
| GDS 5 (N=42) | 32.6% (24.6;41.4) | 16.7% (7.0;31.3) | 83.3% (68.6;93.0) |
| GDS 6 (N=28) | 21.7% (14.9;29.8) | 7.1% (0.9;23.5) | 92.9% (76.5;99.1) |
| GDS 7 (N=13) | 10.1% (5.5;16.6) | 30.8% (9.1;61.4) | 69.2% (38.6;90.9) |
| **Mean number of symptoms per patient** ^2^ | 5.0 (2.4) | 2.3 (2.0) | 3.0 (2.2) |
| **- Sex** |  |  |  |
| Men | 4.9 (2.2) | 1.6 (1.1) | 2.8 (2.3) |
| Women | 5.1 (2.5) | 2.5 (1.1) | 3.0 (2.2) |
| **- Age m (SD)** |  |  |  |
| < 65 years (N=2) | 4.5 (2.1) | 2.2 (1.3) | 2.0 (1.4) |
| 65-74 years (N=21) | 5.3 (2.4) | 2.3 (2.5) | 3.0 (2.6) |
| 75-79 years (N=15) | 4.9 (2.5) | 2.3 (0.8) | 2.9 (2.5) |
| ≥ 80 years (N=91) | 5.0 (2.4) | 2.3 (1.2) | 2.9 (2.1) |
| **- GDS** |  |  |  |
| GDS 3 (N=8) | 4.6 (1.4) | 2.0 (0) | 2.3 (1.5) |
| GDS 4 (N=38) | 5.1 (2.5) | 2.5 (0.5) | 2.6 (1.9) |
| GDS 5 (N=42) | 5.2 (2.4) | 2.7 (1.3) | 3.1 (2.5) |
| GDS 6 (N=28) | 5.1 (2.3) | 2.0 (2.8) | 3.2 (2.1) |
| GDS 7 (N=13) | 4.8 (3.0) | 1.5 (1.0) | 3.1 (2.8) |
| **Percentage of patients based on the number of symptoms** ^1^ |  |  |  |
| 0 | 1.6 (0.2;5.5) | 10.0 (1.2;31.7) | NA |
| 1 | 2.3 (0.5;6.6) | 5.0 (0.1;24.8) | 17.4 (10.8;25.9) |
| 2 | 13.2 (7.9;20.3) | 45.0 (23.1;68.5) | 18.3 (11.6;26.9) |
| 3 | 12.4 (7.3;19.4) | 30.0 (11.9;54.3) | 22.0 (14.6;30.9) |
| 4 | 14.0 (8.5;21.2) | 5.0 (0.1;24.8) | 15.6 (9.4;23.8) |
| 5 | 14.7 (9.1;22.0) | 5.0 (0.1;24.8) | 11.0 (5.8;18.4) |
| 6 | 12.4 (7.3;19.4) | 0 | 7.3 (3.2;13.9) |
| 7 | 10.9 (6.0;17.5) | 0 | 2.8 (0.6;7.8) |
| 8 | 11.6 (6.7;18.5) | 0 | 4.6 (1.5;10.4) |
| 9 | 3.1 (0.9;7.7) | 0 | 0.9 (0;5.0) |
| 10 | 3.9 (1.3;8.8) | 0 | 0 |

1 ［% (patients) (95% CI)]; 2 ［Mean (symptoms) (SD)]
