# Supplementary material for: Neuropsychiatric symptoms and subsyndromes in patients with different stages of dementia in primary care follow-up (NeDEM project): a cross-sectional study
Source: BMC Geriatr. 2022 Jan 22;22:71. doi: 10.1186/s12877-022-02762-9 (PMC8783993; doi:10.1186/s12877-022-02762-9)
Supplement: Supplementary file 2 — Additional file 2. Prevalence of neuropsychiatric symptoms and subsyndromes in patients with dementia included in the study. [file 12877_2022_2762_MOESM2_ESM.docx]

| **Neuropsychiatric symptoms and subsyndromes** | **Total**  **(N = 129)** | | **Significant symptoms¹ (NPI ≥ 4) (N = 109)** | | **Severe symptoms²**  **(N = 59)** | |
| --- | --- | --- | --- | --- | --- | --- |
|  | **n** | **% (95% CI)** | **n** | **% (95% CI)** | **n** | **% (95% CI)** |
| **Symptoms** |  |  |  |  |  |  |
| Elation/euphoria | 22 | 17.1 (11.0;26.7) | 6 | 4.7 (1.7;9.8) | 2 | 1.6 (0.2;5.5) |
| Appetite/eating | 39 | 30.2 (22.5;38.9) | 28 | 21.7 (14.9;29.8) | 10 | 7.8 (3.8;13.8) |
| Aberrant motor behaviour | 40 | 31.0 (23.2;39.7) | 24 | 18.6 (12.3;26.4) | 8 | 6.2 (2.7;11.9) |
| Disinhibition | 46 | 35.7 (27.4;44.6) | 27 | 20.9 (14.3;29.0) | 9 | 7.0 (3.2;12.8) |
| Hallucinations | 49 | 38.0 (29.6;46.9) | 32 | 24.8 (17.6;33.2) | 15 | 11.6 (6.7;18.5) |
| Delusions | 52 | 40.3 (31.8;49.3) | 32 | 24.8 (17.6;33.2) | 14 | 10.9 (6.1;17.5) |
| Anxiety | 56 | 43.4 (34.7;52.4) | 29 | 22.5 (15.6;30.7) | 15 | 11.6 (6.7;18.5) |
| Depression/dysphoria | 61 | 47.3 (38.4;56.3) | 32 | 24.8 (17.6;33.2) | 8 | 6.2 (2.7;11.9) |
| Sleep behaviour | 62 | 48.1 (39.2;57.0) | 30 | 23.3 (16.3;31.5) | 14 | 10.9 (6.1;17.5) |
| Irritability/lability | 63 | 48.8 (39.9;57.8) | 45 | 34.9 (26.7;43.8) | 10 | 7.8 (3.8;13.8) |
| Agitation/aggression | 72 | 55.8 (46.8;64.5) | 44 | 34.1 (26.0;43.0) | 24 | 18.6 (12.3;26.4) |
| Apathy/indifference | 90 | 69.8 (61.1;77.5) | 48 | 37.2 (28.9;46.2) | 16 | 12.4 (7.3;19.4) |
| **Subsyndromes** |  |  |  |  |  |  |
| Hyperactivity | 111 | 86.0 (78.8;91.5) | 81 | 62.8 (53.8;71.1) | 33 | 25.6 (18.3;34.0) |
| Apathy | 100 | 77.5 (69.3;84.4) | 63 | 48.8 (39.9;57.8) | 23 | 17.8 (11.7;25.5) |
| Psychosis | 86 | 66.7 (57.8;74.7) | 57 | 44.2 (35.4;53.2) | 26 | 20.2 (13.6;28.1) |
| Affective | 84 | 65.1 (56.2;73.3) | 47 | 36.4 (28.1;45.4) | 20 | 15.5 (9.7;22.9) |
| ¹ Significant symptoms: those with an NPI frequency by severity score ≥ 4. | | | | | | |
| ² Severe symptoms: those symptoms with a score of 3 in severity, highly bothersome for the patient and difficult for the caregiver to manage. | | | | | | |

Appendix 2 Prevalence of neuropsychiatric symptoms and subsyndromes of patients with dementia included in the study
