# Supplementary material for: Neuropsychiatric symptoms and subsyndromes in patients with different stages of dementia in primary care follow-up (NeDEM project): a cross-sectional study
Source: BMC Geriatr. 2022 Jan 22;22:71. doi: 10.1186/s12877-022-02762-9 (PMC8783993; doi:10.1186/s12877-022-02762-9)
Supplement: Supplementary file 3 — Additional file 3. Intensity of symptoms and neuropsychiatric subsyndromes in patients with dementia included in the study. [file 12877_2022_2762_MOESM3_ESM.docx]

Appendix 3 Intensity of symptoms and neuropsychiatric subsyndromes in patients with dementia included in the study

| **Neuropsychiatric symptoms and subsyndromes** | **Intensity (frequency x severity)¹** | | | |
| --- | --- | --- | --- | --- |
|  | **Total (N=129)** | | **Significant symptoms² (N = 109)** | |
|  | m (SD) | median (IQR) | m (SD) | median (IQR) |
| **Symptoms** |  |  |  |  |
| Elation/euphoria | 0.5 (1.5) | 0 (0-0) | 6.0 (3.1) | 5.0 (4.0-7.5) |
| Appetite/eating | 1.8 (3.4) | 0 (0-1.5) | 7.6 (2.8) | 7.0 (6.0-9.0) |
| Aberrant motor behaviour | 1.5 (2.9) | 0 (0-1.0) | 6.8 (2.8) | 6.0 (4.0-9.0) |
| Disinhibition | 1.7 (3.1) | 0 (0-2.0) | 6.8 (3.2) | 6.0 (4.0-9.0) |
| Hallucinations | 2.2 (3.7) | 0 (0-3.5) | 7.8 (3.1) | 8.0 (4.5-12) |
| Delusions | 2.1 (3.6) | 0 (0-3.5) | 7.6 (3.3) | 7.0 (4.0-12.0) |
| Anxiety | 2.0 (3.3) | 0 (0-2.5) | 7.3 (2.9) | 6.0 (4.0-9.0) |
| Depression/dysphoria | 2.1 (3.2) | 0 (0-3.5) | 7.0 (2.5) | 6.0 (4.5-8.0) |
| Sleep behaviour | 2.3 (3.6) | 0 (0-3.0) | 8.1 (3.0) | 8.0 (5.5-12.0) |
| Irritability/lability | 2.5 (3.4) | 0 (0-4.0) | 6.7 (2.5) | 6.0 (4.0-8.0) |
| Agitation/aggression | 3.2 (4.2) | 1 (0-6.0) | 8.4 (3.2) | 8.5 (6.0-12.0) |
| Apathy/indifference | 3.2 (3.6) | 2 (0-5.0) | 7.0 (2.9) | 6.0 (4.0-9.0) |
| **Subsyndromes** |  |  |  |  |
| Hyperactivity | 9.4 (9.3) | 6.0 (1.5-15) | 10.9 (9.3) | 10.0 (4.0-16.0) |
| Apathy | 5.0 (5.4) | 3.0 (1.0-8.0) | 5.7 (5.4) | 5.0 (1.0-8.0) |
| Psychosis | 6.5 (8.7) | 3.0 (0-10.0) | 7.6 (9.0) | 5.0 (0-12.0) |
| Affective | 4.0 (5.2) | 2.0 (0-6.0) | 4.6 (5.4) | 2.0 (0-8.0) |

| ^1^ Range of F x G from 0 to 12 for all symptoms; range from 4 to 12 for significant symptoms. Not applicable for subsyndromes. | |
| --- | --- |
| ^2^ Significant symptoms: those with an NPI frequency by severity score ≥ 4. |  |
