# Supplementary material for: Neuropsychiatric symptoms and subsyndromes in patients with different stages of dementia in primary care follow-up (NeDEM project): a cross-sectional study
Source: BMC Geriatr. 2022 Jan 22;22:71. doi: 10.1186/s12877-022-02762-9 (PMC8783993; doi:10.1186/s12877-022-02762-9)
Supplement: Supplementary file 4 — Additional file 4. Frequency and intensity of neuropsychiatric symptoms based on the progression of dementia (GDS stage). [file 12877_2022_2762_MOESM4_ESM.docx]

Appendix 4 Frequency and intensity of neuropsychiatric symptoms based on the progression of dementia (GDS stage)

| **Neuropsychiatric symptoms** | **GDS 3 (N = 8)** | | **GDS 4 (N = 38)** | | **GDS 5 (N = 42)** | | **GDS 6 (N = 28)** | | **GDS 7 (N = 13)** | |
| --- | --- | --- | --- | --- | --- | --- | --- | --- | --- | --- |
|  | Frequency | Intensity | Frequency | Intensity | Frequency | Intensity | Frequency | Intensity | Frequency | Intensity |
|  | n (%) | m (SD) | n (%) | m (SD) | n (%) | m (SD) | n (%) | m (SD) | n (%) | m (SD) |
|  |  |  |  |  |  |  |  |  |  |  |
| Elation/euphoria | 2 (25.0) | 1.2 (2.4) | 7 (18.4) | 0.3 (0.9) | 5 (11.9) | 0.3 (1.0) | 7 (25.0) | 1.0 (2.5) | 1 (7.7) | 0.1 (0.6) |
| Appetite/eating | 1 (12.5) | 0.1 (0.3) | 8 (21.1) | 1.2 (2.9) | 16 (38.1) | 2.2 (3.5) | 10 (35.7) | 2.4 (3.9) | 4 (30.8) | 2.2 (4.0) |
| Aberrant motor behaviour | 1 (12.5) | 0.1 (0.3) | 9 (23.7) | 0.7 (1.6) | 16 (38.1) | 2.3 (3.9) | 9 (32.1) | 1.6 (2.8) | 5 (38.5) | 1.4 (2.5) |
| Disinhibition | 1 (12.5) | 0.5 (1.4) | 19 (50.0) | 1.9 (2.9) | 16 (38.1) | 1.9 (2.3) | 8 (28.6) | 1.6 (3.3) | 2 (15.4) | 1.1 (3.4) |
| Hallucinations ^1^ | 1 (12.5) ^1^ | 0.2 (0.7) ^1^ | 12 (31.6) ^1^ | 1.4 (3.0) ^1^ | 13 (31.0) ^1^ | 1.8 (3.5) ^1^ | 17 (60.7) ^1^ | 3.4 (4.0) ^1^ | 6 (46.2) ^1^ | 3.9 (4.8) ^1^ |
| Delusions | 2 (25.0) | 1.0 (1.8) | 14 (36.8) | 1.2 (2.4) | 15 (35.7) | 2.5 (4.4) | 16 (57.1) | 2.5 (3.2) | 5 (38.5) | 3.4 (4.6) |
| Anxiety | 4 (50.0) | 1.5 (3.0) | 18 (47.4) | 1.7 (3.0) | 17 (40.5) | 2.2 (3.5) | 10 (35.7) | 1.8 (3.4) | 7 (53.8) | 2.2 (3.5) |
| Depression/dysphoria | 5 (62.5) | 2.4 (3.3) | 22 (57.9) | 2.1 (3.0) | 20 (47.6) | 2.4 (3.5) | 9 (32.1) | 2.0 (3.4) | 5 (38.5) | 1.3 (2.0) |
| Sleep behaviour | 5 (62.5) | 2.5 (3.1) | 12 (31.6) | 1.9 (3.8) | 20 (47.6) | 1.9 (3.4) | 17 (60.7) | 2.5 (3.4) | 8 (61.5) | 3.7 (4.6) |
| Irritability/lability | 5 (62.5) | 2.6 (2.9) | 22 (57.9) | 2.7 (3.2) | 22 (52.4) | 3.0 (3.6) | 10 (35.7) | 1.7 (3.2) | 4 (30.8) | 2.0 (4.4) |
| Agitation/aggression | 4 (50.0) | 1.0 (1.4) | 21 (55.3) | 2.9 (3.7) | 28 (66.7) | 3.7 (4.4) | 13 (46.4) | 3.8 (5.0) | 6 (46.2) | 2.9 (4.6) |
| Apathy/indifference | 6 (75.0) | 2.4 (3.0) | 28 (73.7) | 2.8 (3.0) | 30 (71.4) | 3.0 (3.3) | 17 (60.7) | 3.5 (4.2) | 9 (69.2) | 4.5 (4.9) |

GDS: Global Deterioration Scale.

^1^ significant association (p < 0.05)
