# Supplementary material for: Neuropsychiatric symptoms and subsyndromes in patients with different stages of dementia in primary care follow-up (NeDEM project): a cross-sectional study
Source: BMC Geriatr. 2022 Jan 22;22:71. doi: 10.1186/s12877-022-02762-9 (PMC8783993; doi:10.1186/s12877-022-02762-9)
Supplement: Supplementary file 5 — Additional file 5. Frequency and intensity of significant neuropsychiatric symptoms (NPI ≥ 4) based on the progression of dementia (GDS stage). [file 12877_2022_2762_MOESM5_ESM.docx]

Appendix 5 Frequency and intensity of significant neuropsychiatric symptoms (NPI ≥ 4) based on the progression of dementia (GDS stage)

| **Neuropsychiatric symptoms** | **GDS 3 (N = 8)** | | **GDS 4 (N = 38)** | | **GDS 5 (N = 42)** | | **GDS 6 (N = 28)** | | **GDS 7 (N = 13)** | |
| --- | --- | --- | --- | --- | --- | --- | --- | --- | --- | --- |
|  | Frequency | Intensity | Frequency | Intensity | Frequency | Intensity | Frequency | Intensity | Frequency | Intensity |
|  | n (%) | m (SD) | n (%) | m (SD) | n (%) | m (SD) | n (%) | m (SD) | n (%) | m (SD) |
| Elation/euphoria | 2 (25.0) | 1.4 (2.5) | 1 (2.6) | 0.4 (0.9) | 1 (2.4) | 0.4 (1.1) | 2 (7.1) | 1.0 (2.5) | 0 | 0.2 (0.7) |
| Appetite/eating | 0 | 0.1 (0.4) | 6 (15.8) | 1.4 (3.0) | 12 (28.6) | 2.6 (3.8) | 7 (25.0) | 2.5 (4.0) | 3 (23.1) | 3.2 (4.5) |
| Aberrant motor behaviour | 0 | 0.1 (0.4) | 4 (10.5) | 0.8 (1.7) | 11 (26.2) | 2.8 (4.1) | 6 (21.4) | 1.7 (2.9) | 3 (23.1) | 1.9 (2.8) |
| Disinhibition | 1 (12.5) | 0.6 (1.5) | 10 (26.3) | 2.2 (3.1) | 10 (23.8) | 2.1 (3.5) | 5 (17.9) | 1.6 (3.4) | 1 (7.7) | 1.7 (4.0) |
| Hallucinations ^1^ | 0^1^ | 0.3 (0.8) ^1^ | 6 (15.8) ^1^ | 1.7 (3.2) ^1^ | 7 (16.7) ^1^ | 2.1 (3.8) ^1^ | 13 (46.4) ^1^ | 3.6 (4.1) ^1^ | 6 (46.2) ^1^ | 5.7 (4.9) ^1^ |
| Delusions | 2 (25.0) | 1.1 (2.0) | 6 (15.8) | 1.5 (2.6) | 10 (23.8) | 2.9 (4.7) | 9 (32.1) | 2.6 (3.3) | 5 (38.5) | 4.9 (4.9) |
| Anxiety | 1 (12.5) | 1.7 (3.3) | 7 (18.4) | 1.9 (3.2) | 12 (28.6) | 2.7 (3.7) | 6 (21.4) | 2.0 (3.5) | 3 (23.1) | 2.8 (4.0) |
| Depression/dysphoria | 2 (25.0) | 2.6 (3.6) | 8 (21.1) | 2.3 (3.2) | 11 (26.2) | 2.7 (3.7) | 8 (28.6) | 2.2 (3.4) | 3 (23.1) | 1.9 (2.3) |
| Sleep behaviour | 3 (37.5) | 2.7 (3.3) | 8 (21.1) | 2.3 (4.0) | 8 (19.0) | 2.2 (3.6) | 7 (25.0) | 2.7 (3.5) | 4 (30.8) | 5.0 (4.9) |
| Irritability/lability | 4 (50.0) | 3.0 (2.9) | 15 (39.5) | 3.1 (3.3) | 18 (42.9) | 3.6 (3.7) | 6 (21.4) | 1.9 (3.3) | 2 (15.4) | 2.8 (5.2) |
| Agitation/aggression | 1 (12.5) | 1.1 (1.5) | 15 (39.5) | 3.4 (3.7) | 15 (35.7) | 4.3 (4.5) | 10 (35.7) | 4.1 (5.1) | 3 (23.1) | 4.2 (5.1) |
| Apathy/indifference | 2 (25.0) | 2.7 (3.0) | 13 (34.2) | 3.2 (3.1) | 16 (38.1) | 3.3 (3.5) | 10 (35.7) | 3.8 (4.2) | 7 (53.8) | 6.3 (4.8) |

GDS: Global Deterioration Scale

^1^ significant association (p < 0.05)
