# Supplementary material for: Neuropsychiatric symptoms and subsyndromes in patients with different stages of dementia in primary care follow-up (NeDEM project): a cross-sectional study
Source: BMC Geriatr. 2022 Jan 22;22:71. doi: 10.1186/s12877-022-02762-9 (PMC8783993; doi:10.1186/s12877-022-02762-9)
Supplement: Supplementary file 6 — Additional file 6. Frequency and intensity of neuropsychiatric subsyndromes based on the progression of dementia (GDS stage). [file 12877_2022_2762_MOESM6_ESM.docx]

Appendix 6 Frequency and intensity of neuropsychiatric subsyndromes based on the progression of dementia (GDS stage)

| **Subsyndromes** | **GDS 3 (N = 8)** | | **GDS 4 (N = 38)** | | **GDS 5 (N = 42)** | | **GDS 6 (N = 28)** | | **GDS 7 (N = 13)** | |
| --- | --- | --- | --- | --- | --- | --- | --- | --- | --- | --- |
|  | Frequency | Intensity | Frequency | Intensity | Frequency | Intensity | Frequency | Intensity | Frequency | Intensity |
|  | n (%) | m (SD) | n (%) | m (SD) | n (%) | m (SD) | n (%) | m (SD) | n (%) | m (SD) |
| Hyperactivity | 7 (87.5) | 5.5 (5.0) | 35 (92.1) | 8.5 (8.2) | 36 (85.7) | 11.2 (10.4) | 22 (78.6) | 9.8 (9.2) | 11 (84.6) | 7.6 (10.2) |
| Apathy | 6 (75.0) | 2.5 (2.9) | 30 (78.9) | 4.0 (5.0) | 34 (81.0) | 5.1 (5.3) | 21 (75.0) | 5.9 (5.4) | 9 (69.2) | 6.7 (7.0) |
| Psychosis | 5 (62.5) | 3.8 (3.8) | 20 (52.6) | 4.6 (7.0) | 29 (69.0) | 6.2 (9.1) | 23 (82.1) | 8.5 (8.4) | 9 (69.2) | 11.0 (12.6) |
| Affective | 7 (87.5) | 3.9 (6.0) | 28 (73.7) | 3.9 (4.6) | 27 (64.3) | 4.6 (5.5) | 15 (53.6) | 3.8 (5.3) | 7 (53.8) | 3.5 (5.3) |

GDS: Global Deterioration Scale
